# Lubrication Performances of Carbon-Doped MoSe_2_ Nanoparticles and Their Biocompatibility Characterization In Vitro

**DOI:** 10.3389/fchem.2020.580151

**Published:** 2021-02-23

**Authors:** Lisha Wang, Tao Hou, Yihong Li, Hailin Lu, Li Gao

**Affiliations:** ^1^ The First Affiliated Hospital of Xi’an Jiaotong University, Health Science Center, Xi’an Jiaotong University, Xi’an, China; ^2^ College of Mechanical & Electronic Engineering, Xi’an Polytechnic University, Xi’an, Shaanxi, China; ^3^ School of Mechanical Engineering, Xi’an Jiaotong University, Xi’an, Shaanxi, China

**Keywords:** carbon-doped, MoSe_2_ nanoparticles, lubrication, biocompatibility, ultrasonic method

## Abstract

Health and environmental protection issues have become major focus areas in many research and development projects. In this context, recent MTT cytotoxicity assessments performed on carbon-doped MoSe_2_ nanoparticles have indicated that they exhibit excellent biocompatibility. Therefore, these nanoparticles have attracted considerable interest from researchers worldwide. Herein, we report the successful synthesis of carbon-doped MoSe_2_ nanoparticles using an ultrasonic method to enhance their lubrication effect for use as oil additives. Carbon-doped MoSe_2_ nanoparticles are smaller than untreated MoSe_2_ nanoparticles and can easily access the contact area to form a tribofilm, reducing the friction coefficient and generating less wear. Moreover, carbon-doped MoSe_2_ nanoparticles and waste water prepared with the nanoparticles display excellent biocompatibility. Hence, they can be used in practical applications such as oil additives.

## Introduction

Molybdenum diselenide (MoSe_2_) is an inorganic material that is part of the family known as transition metal dichalcogenides, which also includes materials such as WS_2_, NbS_2_, MoS_2_, WSe_2_, and many more. These materials are widely used in several fields, such as optoelectronics, medical science, and the manufacture of batteries (Li et al., 2014; Shi et al., 2014). Although MoSe_2_ is not biocompatible, its toxicity is relatively low compared with other two-dimensional transition metal dichalcogenide materials (Pan et al., 2018). Moreover, classic 2D materials such as grapheme and its analogs display higher cytotoxicities than MoSe_2_, and selenium and molybdenum are essential trace elements in a healthy human body (Teo et al., 2014). Therefore, improving the biocompatibility of MoSe_2_ is vital (Chai et al., 2018; Chen et al., 2018; Wu et al., 2018; Kim et al., 2019; Qi and Liu, 2019; Zhang et al., 2019a; Zhang et al., 2019b), and the current research regarding the toxicity of MoSe_2_ nanoparticles has important implications for health and the environment.

Lubrication is important for extending the operational lifetime of machines comprising moving parts. In addition, efficient lubrication saves energy, making it crucial in any mechanical system (Li et al., 2019). Current research is focused on the development of biocompatible, eco-friendly, and efficient lubricating materials (Seyedzavvar et al., 2019). In this context, nanomaterials have garnered significant attention since they have shown outstanding lubricating properties, as well as electrocatalytic, optical, magnetic, and antineoplastic properties (Chen et al., 2016; Wilhelm et al., 2016; Shi et al., 2017). Interestingly, nanomaterials exhibit various anti-friction and anti-wear properties under different conditions, and their preparation methods, morphologies, particle size distribution, dispersibilities, and concentrations, can significantly influence their lubrication effects (Joly-Pottuz et al., 2005; Zhang et al., 2014; Aldana et al., 2016; Dai et al., 2016; Ding et al., 2018). Zhang et al. found that oil containing MoSe_2_ nanoflowers exhibited lower friction coefficients than that of the base oil. The formation of a smooth tribofilm on worn surfaces is believed to be responsible for the excellent lubricating effects (Zhang et al., 2015). Chen et al. synthesized graphene/graphene-like MoSe_2_ (NH_2_-rGO/MoSe_2_) hybrid nanosheets and used them as lubricant additives; the hybrids were doped into a bismaleimide (BMI) matrix to reinforce their mechanical and tribological properties (Chen et al., 2019). Xue et al. revealed that MoSe_2_ hollow nanospheres possessed anti-wear and friction-reducing properties when employed as a lubrication additive. The nanospheres entered the interface with the base oil, and subsequently formed a tribofilm that enhanced the tribological performances (Xue et al., 2017). Notably, the majority of current research focuses on the development of superior lubrication effects, however, almost no study considers the environmental pollution and toxicity of these particles in oil additives.

In this work, an ultrasonic method was used to successfully prepare carbon-doped MoSe_2_ nanoparticles. Scanning electron microscopy (SEM), transmission electron microscopy (TEM), Fourier transform infrared (FT-IR), X-ray photoelectron spectroscopy (XPS), and thermogravimetric (TG) analysis were used to investigate the material properties. The MoSe_2_ nanoparticles were used as an oil additive, and their tribological performances were investigated under different concentrations. The MTT method was used to test the toxicity of the carbon-doped MoSe_2_ nanoparticles and waste water prepared with nanoparticles; the cell viabilities of KH-2, ACHN, and 786-O cells were measured. Due to their exceptional lubrication effect and nontoxicity, these carbon-doped MoSe_2_ nanoparticles are an excellent eco-friendly oil additive.

## Experimental

### Materials

Selenium (Se) powder was purchased from the Beilian Fine Chemicals Development Co., Ltd., (Shanghai, China). Na_2_MoO_4_·2H_2_O (Sodium molybdate dehydrate) was purchased from Chemical Reagent Plant Four (Tianjing, China). N_2_H_4_·H_2_O (Hydrazine hydrate 80%) was purchased from the Fuyu Fine Chemical Co., Ltd., (Tianjing, China). All materials used were analytic reagent grade.

### Preparation

Initial MoSe_2_ powder: 1.53 g of Na_2_MoO_4_·2H_2_O, 1 g of Se powder, 80 ml of distilled water, and 10 ml of N_2_H_4_·H_2_O were added to a 200 ml Teflon-lined stainless-steel autoclave. The mixture was thoroughly stirred, tightly sealed, and maintained at 200°C for 12 h in the hydrothermal reactor. After cooling the system to room temperature, centrifugal washing with deionized water was performed to obtain the product. Finally, the product was dried at 60°C to obtain the initial MoSe_2_ powder.

Undoped MoSe_2_ nanoparticles: 100 ml of distilled water was added in a beaker and the initial MoSe_2_ powder was added to this. The MoSe_2_ nanoparticles were subsequently obtained by performing ultrasonic dispersion for 2 h. The product was dried at 60°C to obtain the undoped MoSe_2_ nanoparticles.

Carbon-doped MoSe_2_ nanoparticles: 50 ml of isopropyl alcohol and 50 ml of distilled water were added to a beaker, and the initial MoSe_2_ powder was subsequently added to this mixture. Carbon-doped MoSe_2_ nanoparticles were obtained by performing ultrasonic dispersion for 2 h. The product was dried at 60°C to obtain carbon-doped MoSe_2_ nanoparticles.

The treated MoSe_2_ nanoparticles were dispersed in white oil using an ultrasonic cleaning machine for 2 h, after which the tribology tests were performed.

### Characterization

FT-IR was performed using a VERTEX70 (Bruker, Germany). XPS was carried out using an AXIS Ultra DLD (Kratos, England). X-ray diffraction (XRD) was performed using a D8 advance (Bruker, Germany). TEM images were acquired using either a JEOL JEM-2100Plus (Japan) or a Bio-TEM Hitachi H-7650 operating at 80 kV. SEM images were acquired using an SU3500 instrument (Techcomp, China). Energy dispersive spectrometer (EDS) was performed using an OXFORD instruments attachment (United Kingdom). Particle size distributions were obtained with a laser particle size distribution analyzer (ZSE, Malvern, United Kingdom). TG analysis and differential scanning calorimetry (DSC) analyses were carried out under an oxygen atmosphere using an STA449F5 (NETZSCH, Germany).

### Tribology Tests

The reciprocation friction wear tests were carried out using a ball-on-disc tribometer (UMT-2, CETR Corporation Ltd., United States). The ball specimens were composed of GCr15 with a diameter of 9.525 mm. The disks were also composed of GCr15 with a diameter of 30 mm, a height of 7 mm, and a hardness of 62–65 HRC. The average surface roughness of the disks was 0.017 μm. All tests were repeated five times to obtain a statistically meaningful average.

### Biocompatible Tests

Human kidney HK-2, 786-O, and ACHN cells were obtained from ATCC (Gibco, United States). The cells were cultured at 37°C with 5% CO_2_ in a humidified atmosphere. The cell experiments were performed in The First Affiliated Hospital of Xi’an Jiaotong University.

## Results and Discussion

### Scanning Electron Microscopy, Transmission Electron Microscopy, and Size Distribution Analyses

**FIGURE 1:**
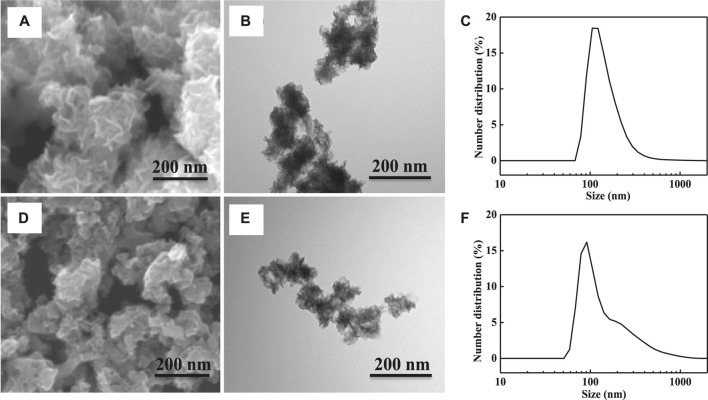
**(A)** SEM image of undoped MoSe_2_ nanoparticles, **(B)** TEM image of undoped MoSe_2_ nanoparticles, **(C)** size distribution of undoped MoSe_2_ nanoparticles, **(D)** SEM image of carbon-doped MoSe_2_ nanoparticles, **(E)** TEM image of carbon-doped MoSe_2_ nanoparticles, and **(F)** size distribution of carbon-doped MoSe_2_ nanoparticles.

Figure 1 shows the morphology and size distributions of the MoSe_2_ nanoparticles. The SEM image in Figure 1A shows that the morphology of the undoped nanoparticles resembles a flower. This is further confirmed by the TEM imaging shown in Figure 1B that also shows the present contrasting layers in the structure. The average size of these nanoparticles was determined to be ∼200 nm, and the majority of the particles were large than 100 nm in size (Figure 1C). Figure 1D shows a SEM image of the carbon-doped nanoparticles; the flower morphology was not observed. In addition, carbon-doped nanoparticles exhibited a smaller average size compared with the undoped particles. The TEM image in Figure 1E and the corresponding size distribution results in Figure 1F indicate that the average size of these nanoparticles was ∼100 nm. This implies that the carbon-doping method is indeed beneficial for enhancing the dispersibility of the nanoparticles.

**FIGURE 2:**
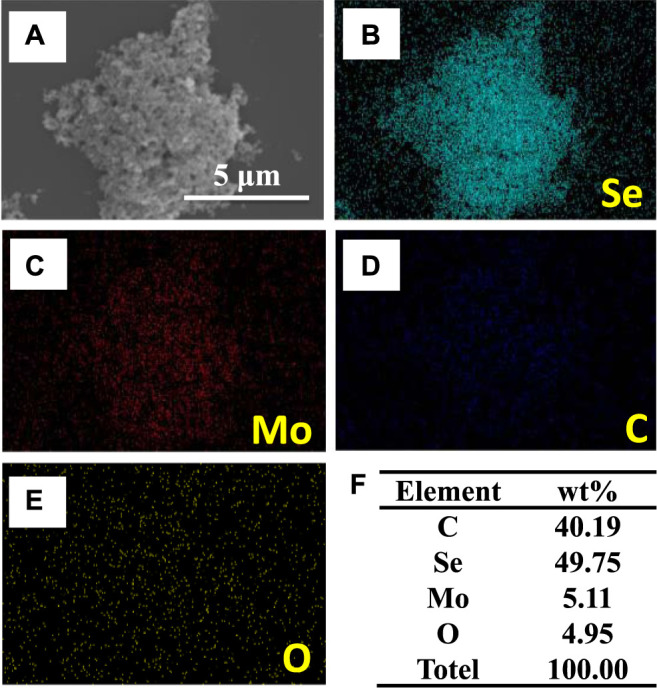
**(A)** SEM image, **(B–E)** EDS elemental maps, and **(F)** derived element wt% in carbon-doped MoSe_2_ nanoparticles.

The carbon-doped MoSe_2_ nanoparticles were further characterized by SEM and EDS elemental mapping. The EDS maps, shown in Figures 2B–E, indicating the presence of C, Se, Mo, and O in the sample. Quantitative elemental analysis is presented in Figure 2F, demonstrating that C is the most abundant element in the system. This carbon originates from the organic solvents employed in the preparation process (ethanol and isopropyl alcohol), where isopropyl alcohol reacts with the nanoparticles during ultrasonication.

### Fourier Transform Infrared, X-Ray Diffraction, X-Ray Photoelectron Spectroscopy, and Thermal Analyses

**FIGURE 3:**
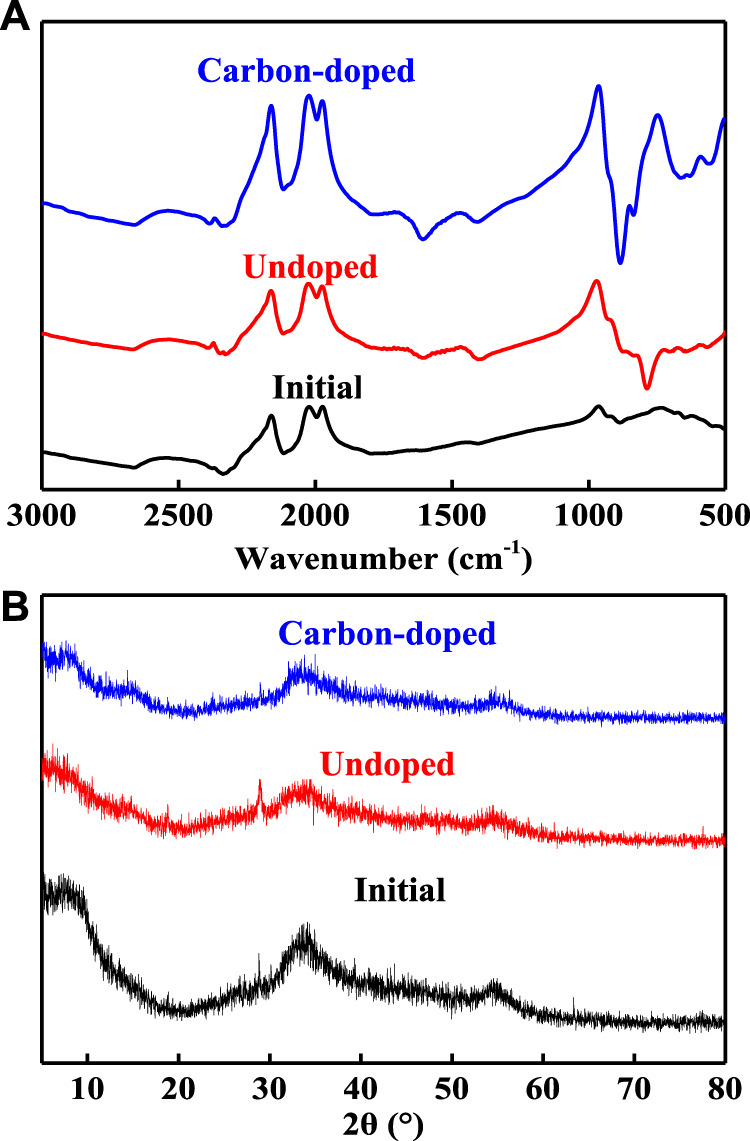
**(A)** FT-IR spectra and **(B)** XRD patterns of the initial, undoped, and carbon-doped MoSe_2_ nanoparticles.

Figure 3A shows the FT-IR spectrum of initial, undoped, and carbon-doped MoSe_2_ nanoparticles. Evidently, the carbon-doped nanoparticles displayed sharp absorption bands. This is because these nanoparticles contained 40.19 wt% carbon, and several carbon functional groups were present. For example, the adsorption band at 656 cm^−1^ was attributed to the C-OH out-of-plane bending in alcohols (Wen, 2010). Moreover, the carbon-doped system exhibited numerous adsorption peaks between 500 and 1,000 cm^−1^, further revealing the existence of abundant carbon functional groups after ultrasound treatment. Figure 3B shows the XRD patterns of the particles. A standard pattern of the MoSe_2_ crystal (JCPDS card, no. 20-0757) was used to analyze the diffraction peaks presented by the nanoparticles. The measured XRD data were in agreement with the standard data (Pan et al., 2018). The XRD patterns of the undoped and carbon-doped MoSe_2_ nanoparticles exhibited broad diffraction peaks, reflecting the nano-size effect of the product (Tang et al., 2014). Hence, FT-IR and XRD data indicated that the carbon-doped MoSe_2_ nanoparticles contained abundant carbon functional groups, and that their structures remained similar to the classic MoSe_2_ crystal.

**FIGURE 4:**
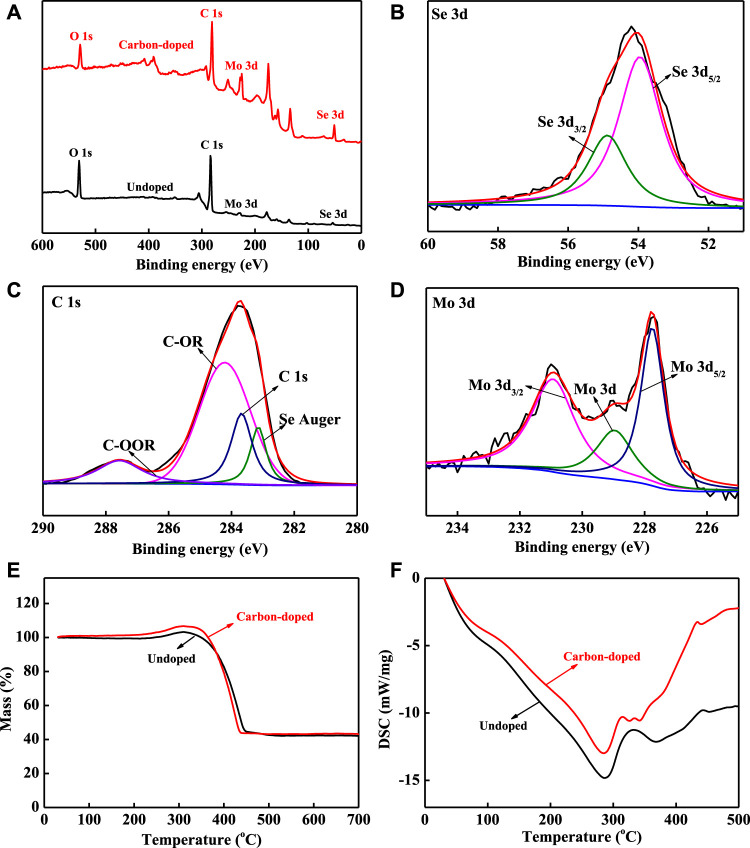
**(A)** XPS survey spectra, **(B–D)** high resolution XPS spectra of carbon-doped MoSe_2_ nanoparticles: **(B)** Se 3d, **(C)** C 1s, **(D)** Mo 3d, **(E)** TG curves, and **(F)** DSC curves.

XPS characterization was employed to analyze the relative composition of the elements and their chemical state in the doped system. Figure 4A shows the presence of O, C, Mo, and Se in the samples, the carbon-doped MoSe_2_ nanoparticles have more distinct characteristic peaks than undoped MoSe_2_ nanoparticles, consistent with previous reports (Wang et al., 2013; Tang et al., 2014; Lei et al., 2015; Xue and Diao, 2017). In Figure 4B, the fitted Se 3d characteristic peaks at 54.9 and 53.9 eV can be attributed to spin-orbital split Se 3d_3/2_ and Se 3d_5/2_, indicating that the Se was present in the Se^2−^ state (Tang et al., 2014; Lei et al., 2015). Figure 4C shows the high resolution XPS spectrum of the C 1s region where four peaks can be fitted: Se Auger (283.2 eV), C 1s (283.7 eV), C–OR (284.2 eV), and COOR (287.5 eV) (Gao et al., 2019). In Figure 4D, the fitted Mo 3d_3/2_, Mo 3d, and Mo 3d_5/2_ peaks are located at 230.9, 228.9 and 227.7 eV (Wang et al., 2013). TG analysis was utilized to study the thermal stability; the two nanoparticle samples exhibited a unique development as shown in Figure 4E. The curves indicate that the weights increased immediately before the temperature reached 311°C, suggesting that the nanoparticles reacted with air at that temperature. The increase was more apparent for carbon-doped MoSe_2_ nanoparticles. The subsequent sharp weight loss in the temperature range of 311–447°C can be attributed to the decomposition of carbon compounds, and the carbon-doped MoSe_2_ nanoparticles plateaued at a lower decomposition temperature. DSC was employed to quantify the total heat of melting (Figure 4F); endothermic peaks for the two nanoparticle samples were observed at 285°C. The curve for the carbon-doped MoSe_2_ nanoparticles appeared above the undoped MoSe_2_ nanoparticles, indicating that the carbon-doped MoSe_2_ nanoparticles required less thermal energy to melt. DSC confirmed that the thermo-oxidative decomposition temperature was approximately 285°C and revealed that the carbon-doped nanoparticles began to react with the air immediately above 285°C. XPS, TG, and DSC demonstrated that the doped nanoparticles contained abundant carbonaceous compounds.

### Tribological Behavior of Carbon-Doped MoSe_2_ Nanoparticles

**FIGURE 5:**
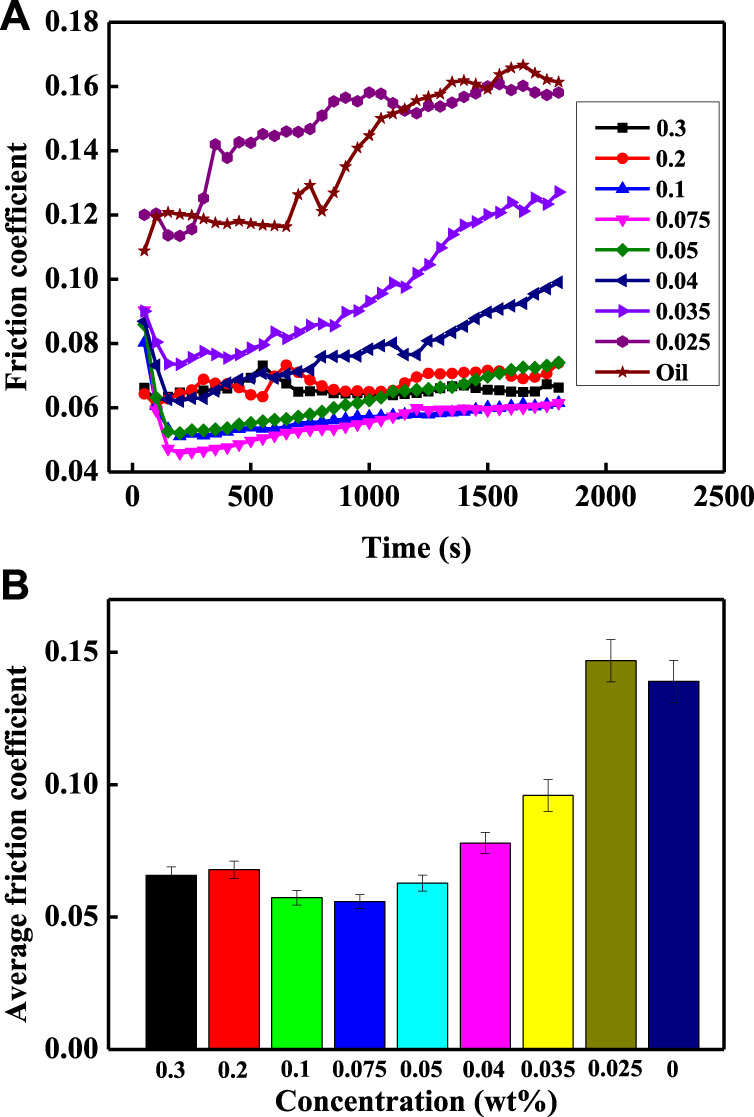
**(A)** Friction coefficients and **(B)** average friction coefficients of carbon-doped MoSe_2_ nanoparticles with different concentrations.

Figure 5A shows the measured friction coefficients for different concentrations of carbon-doped MoSe_2_ nanoparticles added to oil. The friction coefficients significantly decreased upon addition of the doped nanoparticles. The friction coefficients also decreased with an increase in the concentration, and the curves became smoother and stable. The lowest average friction coefficients achieved for carbon-doped nanoparticles was 0.06 (Figure 5B), substantially lower compared with undoped MoSe_2_ nanoparticles (Li et al., 2019). The smaller particles present in the doped system can easily access the contact area, forming a tribofilm. At nanoparticle concentrations of 0.1 wt% and 0.075 wt%, the average friction coefficient was reduced by 59% compared to the pure oil. Moreover, the average friction coefficients were similar when the concentrations were between 0.05 and 0.3 wt%. The average friction coefficient was almost equal to that of pure oil when the concentration was 0.025 wt%, suggesting that the amount of added nanoparticles was too low to significantly alter the lubricating property of the oil. Hence, these results clearly indicate that when carbon-doped MoSe_2_ nanoparticles as used as additives, they can considerably improve the lubrication effect of oil, and the friction processes become smoother and more stable over time.

**FIGURE 6:**
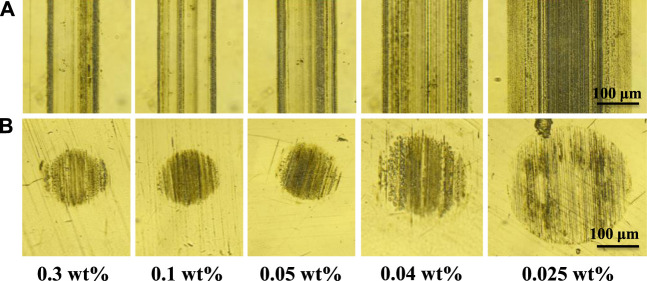
Wear morphology images of **(A)** GCr15 ball surfaces and **(B)** GCr15 disk surfaces for different nanoparticle concentrations.

**FIGURE 7:**
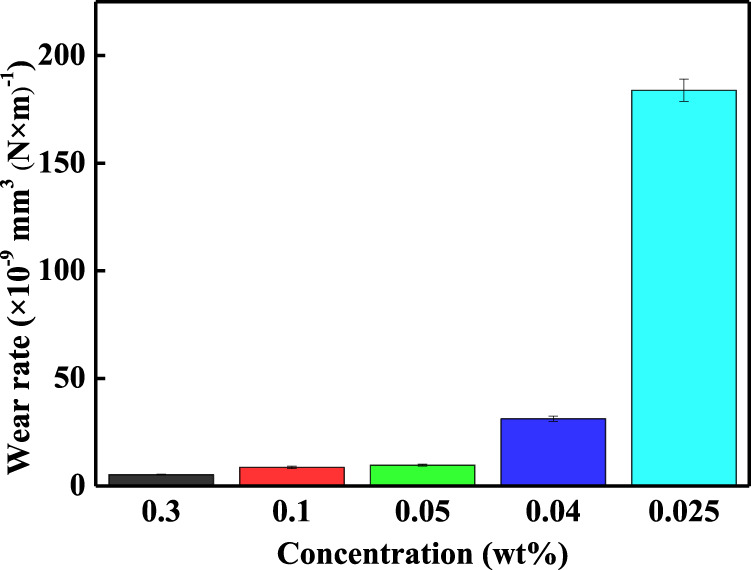
Wear rate under different nanoparticle concentrations.

The worn surfaces of GCr15 disks and balls were investigated using a metallographic microscope and the corresponding images are shown in Figure 6. Figure 6A shows the worn surfaces of GCr15 disks. The width of the wear traces increased from 122.15 to 295.94 μm with a decrease in the concentration. Figure 6B shows the worn surfaces of the GCr15 balls. The worn surfaces displayed significant wear when the nanoparticle concentration was low (0.025 wt%). This is because the quantity of the additive was so low that an anti-friction effect was not generated. The corresponding wear rates of the GCr15 balls are depicted in Figure 7, demonstrating that for an appropriate concentration (0.05 wt%), the carbon-doped MoSe_2_ nanoparticles not only displayed excellent anti-friction properties, but could also impart superior anti-wear capabilities.

### Cell Viability with MoSe_2_ Nanoparticles

**FIGURE 8:**
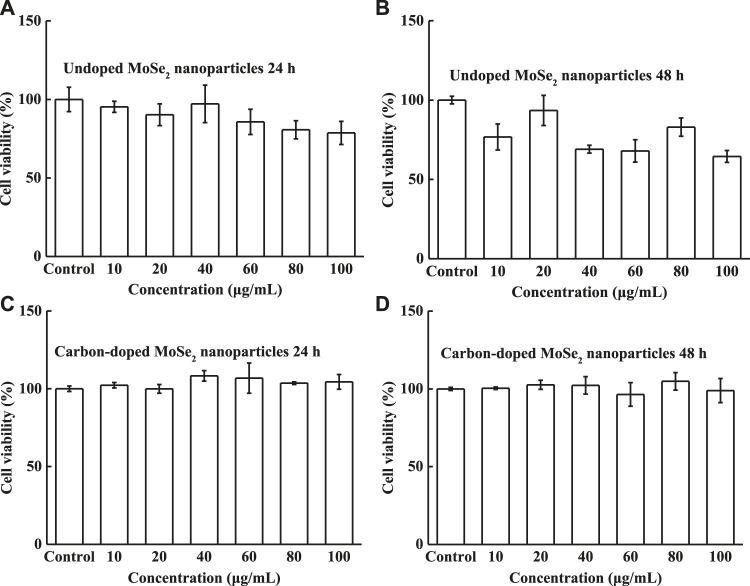
Cell viability of HK-2 cells. **(A)** Undoped MoSe_2_ nanoparticles after incubation for 24 h, **(B)** undoped MoSe_2_ nanoparticles after incubation for 48 h, **(C)** carbon-doped MoSe_2_ nanoparticles after incubation for 24 h, and **(D)** carbon-doped MoSe_2_ nanoparticles after incubation for 48 h with different concentrations.

The undoped and carbon-doped MoSe_2_ nanoparticles inhibited the proliferation of KH-2 cells treated with different concentrations of DMSO (control) for 24 and 48 h. An MTT assay was performed to test the cell viability. Figures 8A,B show the cell viabilities of KH-2 cells with various concentrations of the undoped MoSe_2_ nanoparticles. The cell viability decreased to 64.4% at a concentration of 100 μg/ml after 48 h incubation. Figures 8C,D show that the carbon-doped MoSe_2_ nanoparticles exhibited excellent biocompatibility; the cell viability was almost 100% with different nanoparticle concentrations. The data revealed that the carbon-doped MoSe_2_ nanoparticles displayed superior biocompatibility compared with the undoped MoSe_2_ nanoparticles.

### Cell Viability of Waste Water

**FIGURE 9:**
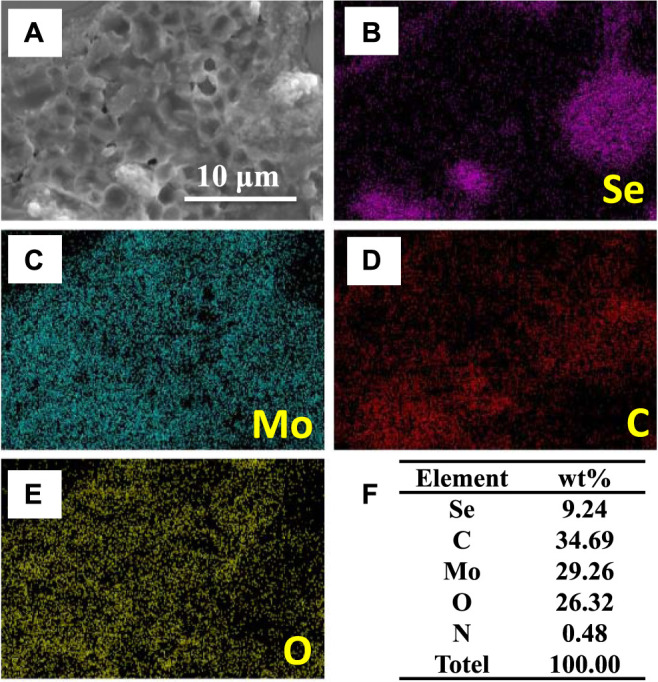
**(A)** SEM image, **(B–E)** EDS elemental maps, and **(F)** element wt% in the dry solid compound.

FT-IR and XPS results showed that the doped particles possessed an abundance of carbon. Therefore, the extent of water pollution must be verified. For this experiment, the carbon-doped nanoparticles were added to distilled water and the solution was ultrasonicated for 1 h. The suspension was then centrifuged to obtain the waste water. Finally, the waste water was evaporated at 60°C to obtain a dry solid compound. EDS elemental mapping was employed to characterize this solid compound. Figure 9A shows that the solid material exhibited a porous morphology, similar to an organic substance. The EDS elemental maps in Figures 9B–E show the presence of C, Se, Mo, and O, and Figure 9F indicates that the amount of oxygen was significantly higher than that found in the carbon-doped MoSe_2_ nanoparticles.

**FIGURE 10:**
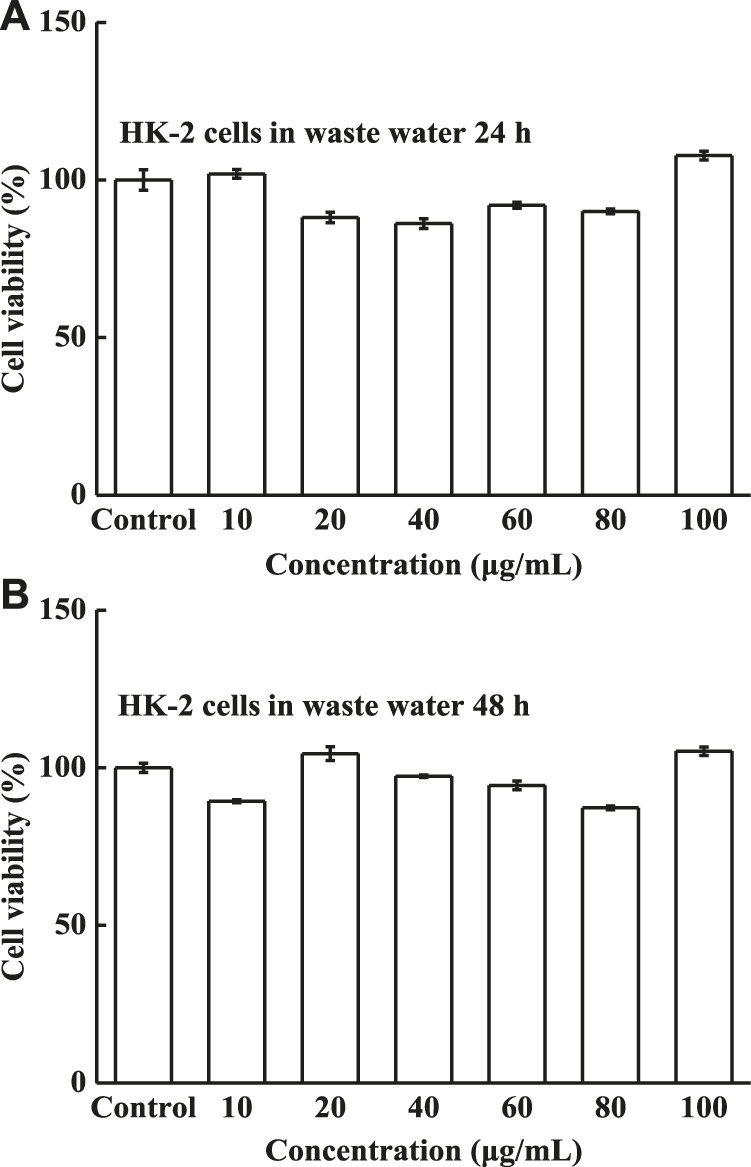
Cell viability of HK-2 cells, **(A)** waste water after incubation for 24 h and **(B)** waste water after incubation for 48 h with different waste water concentrations.

**FIGURE 11:**
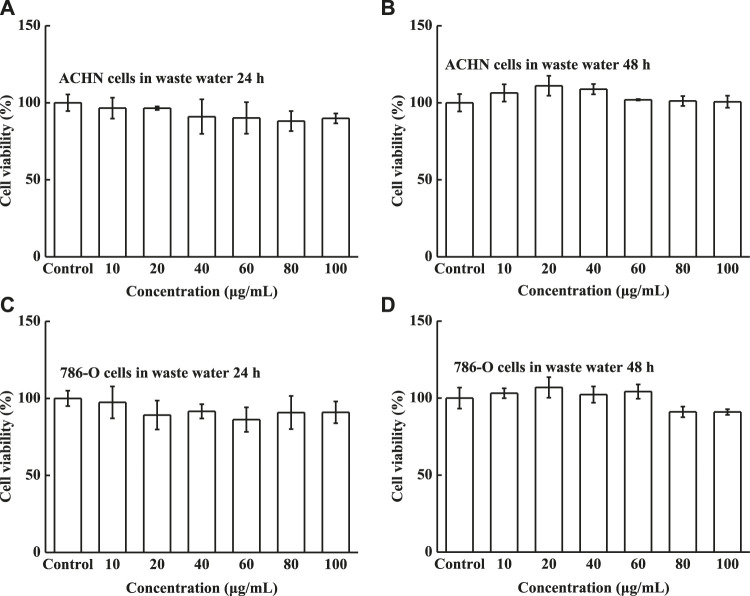
Cell viability of **(A)** ACHN cells in waste water for 24 h, **(B)** ACHN cells in waste water for 48 h, **(C)** 786-O cells in waste water for 24 h, and **(D)** 786-O cells in waste water for 48 h under different concentrations.

The solid compound was diluted with distilled water to obtain waste water with different concentrations, and the cytotoxicity, as a function of the concentration, was subsequently evaluated using the MTT assay. The cell viabilities of HK-2 cells were measured after 24 and 48 h incubation with various concentrations of waste water. The waste exhibited excellent biocompatibility; all cell viabilities were above 87% (Figure 10), and the cell viabilities became stable with increasing concentration. The cell viabilities of ACHN and 786-O cells are shown in Figure 11, verifying that the two human renal carcinoma cell lines also displayed high viability after incubation with waste water for 24 and 48 h. *In vivo* cells retained high viabilities, however, cells easily die *in vitro*. *In vitro* waste water did not kill the ACHN and 786-O cells, indicating that it is impossible to kill cells *in vivo*. These results therefore proved that the waste water is safe for HK-2, ACHN and 786-O cells *in vitro*, and even safer *in vivo*.

### Relevant Mechanisms

**FIGURE 12:**
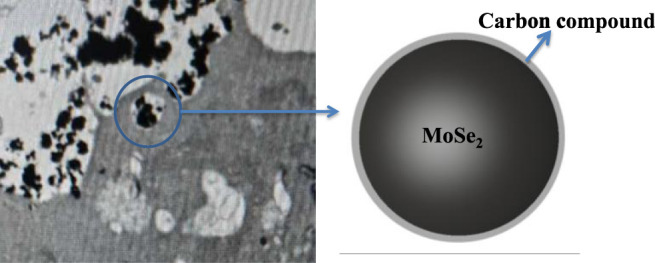
Cytophagy of the carbon-doped MoSe_2_ nanoparticles.

The isopropyl alcohol employed in the preparation process reacted with the nanoparticles when ultrasonic vibrations were applied. In particular, the vibrations produced ultrasonic cavitation phenomenon where large amounts of energy were released through cavitation bubbles, generating high local temperatures and pressures in the environment at the moment of bubble rupture (Feng et al., 2002). These conditions led to chemical bond breakage, water phase combustion, or thermal decomposition of the organic mixture (isopropyl alcohol/distilled water) inside the cavitation bubble (Worapun et al., 2009). This promoted the formation of carbide that covered the outer surface of the nanoparticles. The FT-IR and XPS data demonstrated that the carbon-doped particles were rich in C-OH and COOR, suggesting that these particles experience hydrogen bonding in aqueous solution (Lu et al., 2017a; Lu et al., 2017b; Lu et al., 2018). The carbon-doped MoSe_2_ nanoparticles bonded with one another through hydrogen bonding, forming larger particles, as shown in Figure 12. This figure also shows that the ACHN cells could uptake a small number of nanoparticles since the outer nanoparticle shell consisted of numerous carbon compound functional groups, improving their biocompatibility and allowing high cell viability *in vitro*.

The carbon-doped MoSe_2_ nanoparticles could easily enter the friction interface as a result of their nano size. Furthermore, the nanoparticles possessed larger surface areas and surface energies, allowing easier entrance into the contact area and adsorption on friction surfaces. As such, the tribofilm formed is beneficial for reducing friction and wear. In addition, the surfaces of carbon-doped MoSe_2_ nanoparticles possessed abundant organic functional groups such as C-OH, C-OR, and COOR and these could bond together to form a network structure (Xing, 2005). This type of agglomeration is called soft-agglomeration, and it allows the nanoparticles to be easily re-dispersed in white oil (Moshkovith et al., 2006). The bonded network structure may also improve particle adsorption onto the contact areas, conducive to the formation of a tribofilm.

## Conclusion

In this study, high power ultrasound was used to produce doped and undoped MoSe_2_ nanoparticles, where pyrolysis and ultrasonic cavitation were the key formation mechanisms. The nanoparticles were studied using several methods, such as EDS, FT-IR, XPS, TG, and DSC. The tribology results proved that the addition of carbon-doped MoSe_2_ nanoparticles to oil significantly reduced its friction coefficient. The carbon-doped MoSe_2_ nanoparticles could enter oil contact surfaces to form a tribofilm, enhancing the tribological performances. Moreover, the MTT results revealed that the carbon-doped MoSe_2_ particles exhibited excellent biocompatibility. Additionally, waste water produced from the nanoparticles also retained a high *in vitro* cell viability, indicating that it is even safer *in vivo*. As a result, these biocompatible carbon-doped MoSe_2_ nanoparticles can be used in practical applications such as additives in oil.

## Data Availability

The raw data supporting the conclusion of this article will be made available by the authors, without undue reservation.
